# Oxygen saturation in intraosseous sternal blood measured by CO-oximetry and evaluated non-invasively during hypovolaemia and hypoxia – a porcine experimental study

**DOI:** 10.1007/s10877-023-00980-z

**Published:** 2023-02-14

**Authors:** Erik Näslund, Lars-Göran Lindberg, Gunnar Strandberg, Catharina Apelthun, Stephanie Franzén, Robert Frithiof

**Affiliations:** 1 Department of Surgical Sciences, Section of Anaesthesia and Intensive Care, Uppsala University, Uppsala, Sweden; 2 Centre for Research & Development, Uppsala University/Region Gävleborg, Gävle, Sweden; 3 Department of Biomedical Engineering, Linköping University, Linköping, Sweden; 4 Department of Anaesthesia, Gävle Hospital, 801 87 Gävle, Sweden

**Keywords:** Sternum, Oximetry, Hypoxia, Hypovolaemia, Pig

## Abstract

Purpose: This study intended to determine, and non-invasively evaluate, sternal intraosseous oxygen saturation (SsO_2_) and study its variation during provoked hypoxia or hypovolaemia. Furthermore, the relation between SsO_2_ and arterial (SaO_2_) or mixed venous oxygen saturation (SvO_2_) was investigated. Methods: Sixteen anaesthetised male pigs underwent exsanguination to a mean arterial pressure of 50 mmHg. After resuscitation and stabilisation, hypoxia was induced with hypoxic gas mixtures (air/N_2_). Repeated blood samples from sternal intraosseous cannulation were compared to arterial and pulmonary artery blood samples. Reflection spectrophotometry measurements by a non-invasive sternal probe were performed continuously. Results: At baseline SaO_2_ was 97.0% (IQR 0.2), SsO_2_ 73.2% (IQR 19.6) and SvO_2_ 52.3% (IQR 12.4). During hypovolaemia, SsO_2_ and SvO_2_ decreased to 58.9% (IQR 16.9) and 38.1% (IQR 12.5), respectively, p < 0.05 for both, whereas SaO_2_ remained unaltered (p = 0.44). During hypoxia all saturations decreased; SaO_2_ 71.5% (IQR 5.2), SsO_2_ 39.0% (IQR 6.9) and SvO_2_ 22.6% (IQR 11.4) (p < 0.01), respectively. For hypovolaemia, the sternal probe red/infrared absorption ratio (SQV) increased significantly from baseline (indicating a reduction in oxygen saturation) + 5.1% (IQR 7.4), p < 0.001 and for hypoxia + 19.9% (IQR 14.8), p = 0.001, respectively. Conclusion: Sternal blood has an oxygen saturation suggesting a mixture of venous and arterial blood. Changes in SsO_2_ relate well with changes in SvO_2_ during hypovolaemia or hypoxia. Further studies on the feasibility of using non-invasive measurement of changes in SsO_2_ to estimate changes in SvO_2_ are warranted.

## Introduction

In emergency and perioperative medicine, it is of utmost importance to have reliable measurements of arterial oxygen saturation to ensure adequate delivery of oxygen to the tissues [1, 2]. A standard method for arterial oxygen saturation monitoring is to use a peripheral pulse oximeter. Although the technique is well established, it is not without problems. The algorithm used for the calculation of arterial oxygen saturation relies on the detection of a pulse wave, potentially impairing peripheral measurements during low perfusion states [3–6]. Non-invasive technology for monitoring respiratory status has several obvious benefits, i.e. easy, pain-free application and ability for continuous monitoring. In recent years, this has led to the development of several new non-invasive applications, such as blood volume measurements, organ viability and estimation of vascular tone [7].

Deep penetration of light into the tissues is required for non-invasive measurement of intraosseous blood flow. The depth of penetration depends on the geometry of the sensor, the chosen wavelength and the intensity of emitted light [8–11]. Numerous publications have demonstrated the possibility of non-invasive blood flow measurement in the bone tissue in various locations, e.g. patella, tibia, clavicle and sternum [8, 9, 11–14]. The sternum has a central anatomical location and a high degree of vascularisation. Also, it has a retained haematopoietic activity with a demand for adequate perfusion, which makes it of clinical interest for non-invasive measurements. We recently demonstrated that a novel non-invasive photoplethysmography (PPG) sternal probe could rapidly and accurately monitor arterial oxygen saturation changes during gradually increasing hypoxia in man [11]. Still, even if data shows a good correlation between sternal probe readings and arterial oxygen saturation (SaO_2_) during hypoxia, it is not completely clear if the probe measures arterial- and/or venous saturation as both are expected to decrease during hypoxia. Earlier publications have estimated sternal intramedullary oxygen saturation (SsO_2_) between 70 and 80%, indicating a mixed arteriovenous oxygen saturation [13, 15]. How low perfusion states affect the blend of arterial and venous blood in the sternum is unknown. In order to conduct and correctly interpret non-invasive measurements of sternal intraosseous oxygen saturation, it is crucial to describe possible perturbations of sternal oxygen delivery in relation to changes in systemic circulation or respiration.

The study was constructed with two separate aims. The first was to determine oxygen saturation in the sternum, using CO-oximetry, during changes in inspired oxygen content and tissue oxygen extraction and its relation to SaO_2_ and mixed venous oxygen saturation (SvO_2_). The primary outcome was SsO_2_ evaluated in anaesthetised pigs during a reduced fraction of inspired oxygen (FiO_2_) or blood loss, increasing oxygen extraction but leaving arterial oxygen saturation unchanged. For this aim, we hypothesised that SsO_2_ would decrease during hypovolaemia and hypoxia.

For the second aim, we wanted to compare sternal medullary-, arterial- and SvO_2_ with non-invasive measurements of changes in intraosseous oxygen saturation, using the earlier described [11] novel sternal probe. The outcome for the second aim was the sternal probe quotient value (SQV) obtained from the sternal probe. We hypothesised that the sternal probe accurately detects decreased SsO_2_. In addition, we hypothesised that SsO_2_ is related to SvO_2_ and that the probe thus will be able to monitor changes in SvO_2_.

## Materials and methods

### Animals

The Animal Ethics Committee of Uppsala University, Sweden, approved the experiment (5.8.18–02325/2019, date of approval 2019-03-29). All animals were treated according to the Swedish Board of Agriculture guidelines and complied with the European Convention for the Protection of Vertebrate Animals used for Experimental and other Scientific Purposes (Council of Europe No 123, Strasbourg 1985). The manuscript adheres to the ARRIVE 2.0 guidelines.

A local farmer in Uppsala, Sweden, supplied male Norweigian Landrace breed/Hampshire/Yorkshire pigs. The animals were born on the farm and housed in large cages with water and food *ad libitum*. Sixteen pigs aged 3–4 months were included in the study.

### Anaesthesia and preparation

Upon arrival from the local breeder (20 min transport), all animals were calm and normothermic. The pigs arrived at 8.00 am at the laboratory in separate large cases, two at a time. Two animals per day were included in the study. All animals were anaesthetised with an intramuscular injection of 6 mg/kg tilétamine-zolezepam (Zoletil^®^, Virbac, Denmark) and 2.2 mg/kg xylazine (Rompun^®^, Elanco, Denmark). The animals were placed on a surgical table and tracheotomised. Mechanical ventilation was started with a Servo-I ventilator (Maquet Critical Care, Solna, Sweden). Ventilator settings was - FiO_2_ 0.3, PEEP 5 cm H_2_0, tidal volume of 10 ml/kg, respiratory rate 25/min. After peripheral venous access had been established in both ears, a bolus dose of 20 mg of morphine and 100 mg ketamine was administered intravenously. Maintenance of general anaesthesia was performed with an infusion of pentobarbital 8 mg/kg/h and morphine 0.26 mg/kg/h dissolved in a buffered solution of Glucose 2.5% 4 ml/kg/h. A separate infusion pump administered rocuronium bromide (Esmeron®, Merck & Co. Inc., Kenilworth, NJ, USA) at 3.6 mg/kg/h. Ringer’s Acetate was administered intravenously at 10 ml/kg/h for the first hour and then reduced to 5 ml/kg/h. During the experiment, anaesthetic depth and pain sensation was monitored by continuous monitoring of mean arterial pressure (MAP), and intermittent evaluation of eyelid reflexes and pain sensation in the hoof.

The animals were surgically prepared with an arterial catheter placed in a right cervical artery. A 3-lumen central venous catheter was inserted through a right-sided cervical vein and forwarded to the central vena cava. The same vessel was used to introduce a ballon tipped pulmonary artery catheter (7.5 F Swan-Ganz, Edwards Lifesciences, Irvine, CA). On the left side of the neck, a central bleeding catheter (an introducer used for Swan-Ganz catheters) was introduced in a cervical vein. A urinary catheter was introduced by a small vesicotomy after a mini-laparotomy. Doppler flow probes were applied to the left renal vein and left carotid artery for a separate study investigating renal function, which is reported elsewhere [16].

**Fig. 1:**
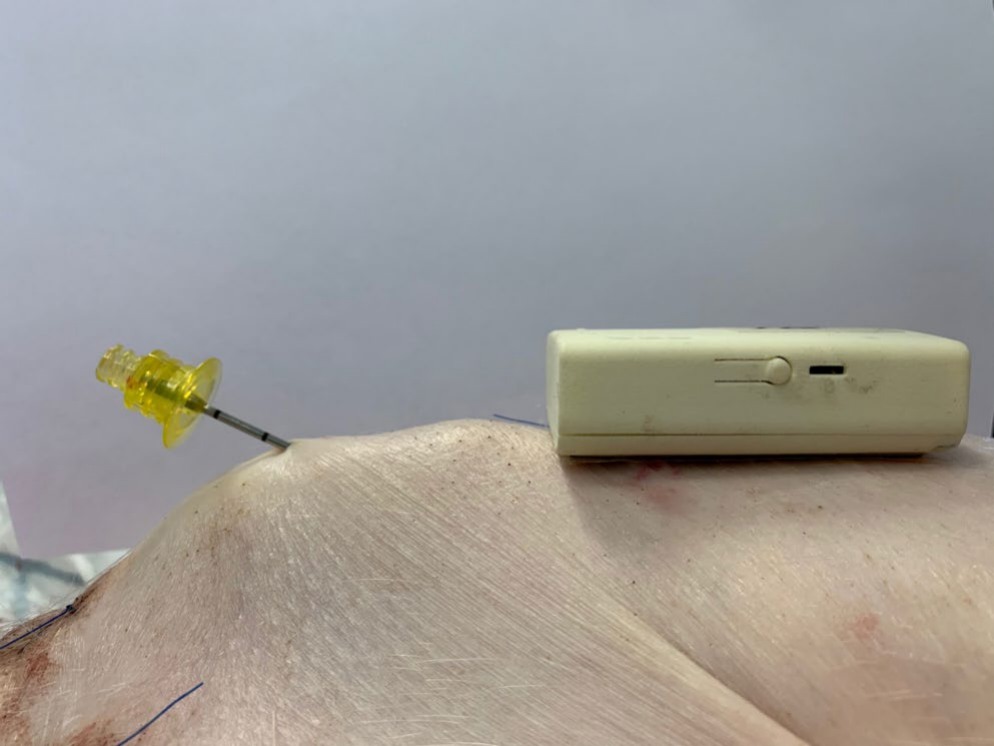
A post-mortem picture illustrating the cranial site of sternal intraosseous cannulation and the caudal placement of the non-invasive sternal probe

After thorough cleansing with soap, water, and ethanol, double adhesive tape was used to attach the sternum probe (RespiHeart) [11] to the caudal part of the sternum. In the cranial part of the sternum, a 45 mm, 15 G intraosseous cannula (EZ-IO, Teleflex corp., Morrisville, NJ) was inserted at approximately 30–45° (Fig. 1). The correct intraosseous placing was verified by aspiration of blood and by post mortem incision down to the bone. All animals were given 60 min to recover before the experimental protocol commenced.

### Protocol

Haemorrhage resulting in hypovolaemia was used to cause hypoperfusion and increased peripheral oxygen extraction. Reduced FiO_2_ was used to lower arterial oxygen saturation. Thus, low sternal medullary oxygen saturation was induced either by increased oxygen extraction or low oxygen delivery. The animals were put in a left lateral position. The protocol started with a 30 min baseline registration, after which bleeding to a predetermined MAP of 50 mmHg was commenced. This MAP-level was kept for 30 min. Blood was collected in heparinised bags. Subsequent resuscitation was done with blood and Ringers Acetate in a 1:1 ratio. Hypoxia was achieved by changing the air supply to the ventilator with nitrogen. An end-tidal O_2_ level of approximately 14–16% was sufficient to produce stable hypoxia of 80% arterial saturation measured by a pulse oximeter.

Blood was sampled from the systemic artery, pulmonary artery and the sternum at baseline, MAP-target, end of bleeding, before and after hypoxia. Marrow blood samples, from the sternum, were analysed spectrophotometrically on an OSM3^®^ blood gas analyser (Radiometer AS, Copenhagen, Denmark). Systemic and pulmonary artery blood samples were analysed both on an ABL800 blood gas analyser (Radiometer AS, Copenhagen, Denmark) and on the OSM3^→^. The OSM3^→^ corrected the readings for porcine haemoglobin. Recordings from the sternal probe were performed before and during the different provocations of hypovolaemia and hypoxia.

At the end of the experiment, the animals were euthanised with an overdose of KCl. Included humane endpoints in the study protocol were; hyperthermia (> 40 °C) at arrival to the laboratory, MAP < 40 mmHg despite treatment, Pulmonary artery pressure > arterial pressure despite treatment and arterial oxygen saturation < 85% despite treatment (besides hypoxic provocation).

### Sternal probe data

The sternum probe has been described in detail previously [11, 17]. The raw signal of the infrared and red light from the sternal probe was transferred via Bluetooth communication to a laptop via a data acquisition programme. Matlab R2018b (Mathworks, Massachusetts, USA) was used for applying a 1 Hz low pass filter and for further analysis of the signal. A quotient of infrared and red lights was formed (Sternal Probe Quotient value – SQV) and subsequently used for comparison against measured SaO_2_, SsO_2_ and SvO_2_.

### Statistics

Since this is the first study using the sternum probe on pigs, no *a priori* sample size calculation could be performed. Data are presented as mean (SD) or median (IQR) for normally and non-normally distributed data, respectively.

Depending on data distribution, a paired t-test or Wilcoxon signed-rank test was used to analyse the differences within the variables between baseline and provocation for normally and non-normally distributed data, respectively. The sampled blood gases were not normally distributed. To analyse the dependent oxygen saturation data between baseline and provocation, for both hypoxia and hypovolaemia, a generalised estimated equation (GEE)-model was estimated assuming an exchangeable correlation structure. The models included an interaction effect between the variables. GEE is suitable for longitudinal analyses of non-normal data [18]. Results from the GEE model are presented as estimated coefficients with the associated standard error (SE). Assessing agreement between blood gas analysis of S_s_O_2_ and S_v_O_2_ was achieved with Bland-Altman plots [19], with data presented as bias (SD). Statistica 13 software (TIBCO Software inc, Palo Alto, CA) was used for analysis and Sigmaplot 14 (Systat Software Inc, San Jose, CA) for graphics. The GEE models were estimated in R version 4.1.1 (R Core Team (2021). R: A language and environment for statistical computing. R Foundation for Statistical Computing, Vienna, Austria. URL https://www.R-project.org/) using the geepack package. A two-sided p-value of < 0.05 was considered statistically significant.

## Results

All animals (n = 16), with a mean weight of 25.8 kg (SD 2.1), completed the experimental protocol. Because of an unstable signal during the hypoxic provocation, sternal probe measurements from one animal had to be excluded during the hypoxic provocation. A total of 170 arterial, sternal and mixed venous blood gases were sampled. Because of technical failure of the blood gas apparatus, three arterial, four sternal and fifteen mixed venous samples could not be analysed.

Four sternal blood samples are missing because of difficulties in the aspiration of blood. Three sternal samples had an aspiration time longer than desired with disproportionate high oxygen saturation and hence were excluded.

Before the commencement of the different provocations, the pigs were normotensive and had a normal arterial oxygen saturation measured by pulse oximetry (SpO_2_) (Table 1). For the hypovolaemic provocation, haemorrhage were successfully used to induce a low circulatory state, and all animals reached the desired MAP-target. During the hypoxic provocation, blood was sampled at baseline and after a steady state of 80% oxygen saturation, as measured by a peripheral pulse oximeter (Table 1). The fraction of inspired oxygen (FiO_2_) required to reach a steady state of 80% oxygen saturation was between 0.14 and 0.18. Some animals developed tachycardia, which was most likely a physiological response to hypoxia, as responses to pain and reflexes were otherwise absent.

**Table 1:** Physiological variables measured during the different provocations

	Normovolaemia	Hypovolaemia	p	Normoxia	Hypoxia	p
Pigs (N)	16	16		15	15	
SpO_2_^a^ (%)	100(IQR 2.0)	100(IQR 0.0)	0.089	100(IQR 0.3)	76(IQR 5.5)	0.002
MAP^b^ (mmHg)	95(IQR 9.0)	50(IQR 2.3)	0.002	88(IQR 10.0)	81(IQR 16.5)	0.014
HR^c^ (beat/min)	88(IQR 15.3)	95.5(IQR 14.3)	0.062	101(IQR 27.3)	136(IQR 32.0)	0.006
CO^d^ (L/min)	3.3(SD 0.9)	2.5(SD 0.4)	0.002			
CVP^e^ (cm H_2_O)	5(IQR 0.5)	1(IQR 1.0)	< 0.001			
PA^f^ (mmHg)	16.9(SD 3.0)	11.7(SD 2.1)	< 0.001			
PCWP^g^ (mmHg)	7.7(SD 1.5)	4.6(SD 1.5)	< 0.001			
SaO_2_^h^ (%)	97.0(IQR 0.3)	97.0(IQR 0.4)	0.44	97.0(IQR 0.2)	71.5(IQR 5.2)	0.001
SsO_2_^i^ (%)	72.0(IQR 7.5)	58.9(IQR 16.)	< 0.001	73.2(IQR 19.6)	39.0(IQR 6.9)	< 0.001
SvO_2_^j^ (%)	55.2(IQR 16.1)	38.1(IQR 12.5)	0.002	52.3(IQR 12.4)	22.6(IQR 11.4)	0.002

^a^Saturation by peripheral pulse oximetry, ^b^Mean Arterial Pressure, ^c^Heartrate, ^d^Cardiac output, ^e^Central venous pressure, ^f^Pulmonary artery pressure, ^g^Pulmonary capillary wedge pressure, ^h^Arterial oxygen saturation, ^i^Sternal intramedullary oxygen saturation, ^j^Mixed venous oxygen saturation. Data are presented as mean (SD) or median (IQR) for normally and non-normally distributed data

### Aim 1 – alterations in SsO_2_

**Fig. 2:**
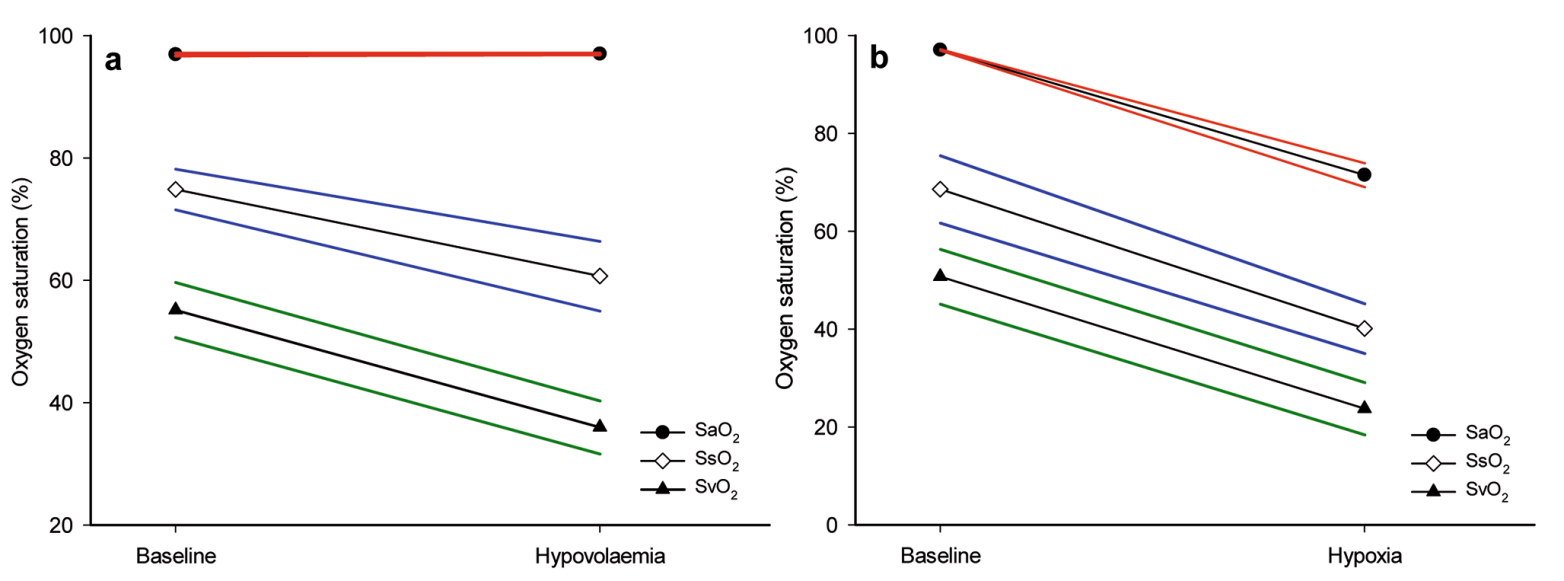
Predicted oxygen saturation (%) values from the Generalised Estimated Equation (GEE) model for hypovolaemia (a) and hypoxia (b), respectively. Standard Errors (SE) are illustrated with red, blue and green lines for arterial (SaO_2_), sternal (SsO_2_) and mixed venous oxygen saturation (SvO_2_), respectively

During the hypovolaemic provocation, SsO_2_ and SvO_2_ decreased, whereas SaO_2_ remained the same (Table 1). The GEE-models tested the interrelationships between SaO_2_, SsO_2_ and SvO_2_. The model’s intercept was 97.0% (SE 0.12). The estimated general difference in oxygen saturation between SsO_2_ versus SaO_2_ and SvO_2_ versus SaO_2_ was − 22.1% (SE 1.70), p < 0.001 and − 41.8% (SE 2.29), p < 0.001, respectively. Hypovolaemia had no significant effect on perturbating the model compared to baseline, + 0.1% (SE 0.14), p = 0.51. The arterial oxygen saturation remained stable throughout the hypovolaemic provocation, but SsO_2_ versus SaO_2_ decreased with − 14.3% (SE 3.37), p < 0.001 and SvO_2_ versus SaO_2_ -19.3% (SE 3.19), p < 0.001, respectively (Fig. 2a).

Contrary to the induced hypovolaemia, during the hypoxic provocation, all oxygen saturations (SaO_2_, SsO_2_ and SvO_2_) decreased significantly (Table 1). The GEE-model’s intercept was 97.1% (SE 0.07). The general average estimated difference between SsO_2_ versus SaO_2_ and SvO_2_ versus SaO_2_ was − 28.5% (SE 3.51), p < 0.001 and − 46.3% (SE 2.86), p < 0.001, respectively. Inducing hypoxia resulted in an average decrease in oxygen saturations with − 25.6% (SE 1.24), p < 0.001. At hypoxia, all evaluated oxygen saturations had decreased, which led to the estimated difference between SsO_2_ and SvO_2_ versus SaO_2_ was no longer found to be significant. SsO_2_ versus SaO_2_ was − 2.9% (SE 4.53), p = 0.53, and SvO_2_ versus SaO_2_ was − 1.4% (SE 4.14), p = 0.74, respectively (Fig. 2b).

### Aim 2 – SQV and SvO_2_

**Fig. 3:**
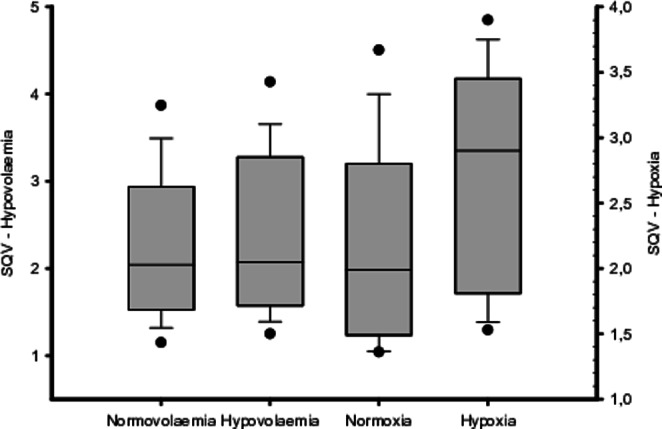
A box plot illustrating the distribution of the sternal probe absorption ratio between infrared and red lights (sternal probe quotient value (SQV)) during baseline and provocation for hypovolaemia and hypoxia, respectively

During both hypovolaemia and hypoxia, SQV increased significantly from baseline, reflecting a measured decrease in oxygen saturation. However, the increase in SQV was not equal for the hypovolaemic and the hypoxic provocation + 5.1% (IQR 7.4), p = 0.001 versus + 19.9% (IQR 14.8), p < 0.001, respectively (Fig. 3).

**Fig. 4:**
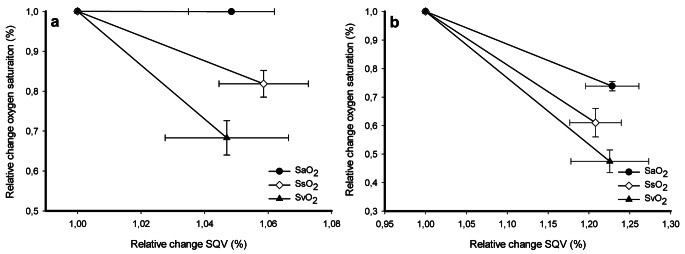
Relative changes between baseline and provocation for oxygen saturation measured in arterial (SaO_2_), sternal (SsO_2_) or mixed venous (SvO_2_) blood plotted against relative changes of the sternal probe quotient value (SQV) during (a) hypovolaemia (SaO_2_ n = 15, SsO_2_ n = 14 SvO_2_ n = 10) and (b) hypoxia (SaO_2_ n = 13, SsO_2_ n = 15, SvO_2_ n = 9). Data are presented as mean (standard error (SE))

Figure 4 illustrates the increase in SQV with the concomitant alterations in SaO2, SsO_2_ and SvO_2_ as the effect of hypovolaemia (Fig. 4a) and hypoxia (Fig. 4b), respectively.

**Fig. 5:**
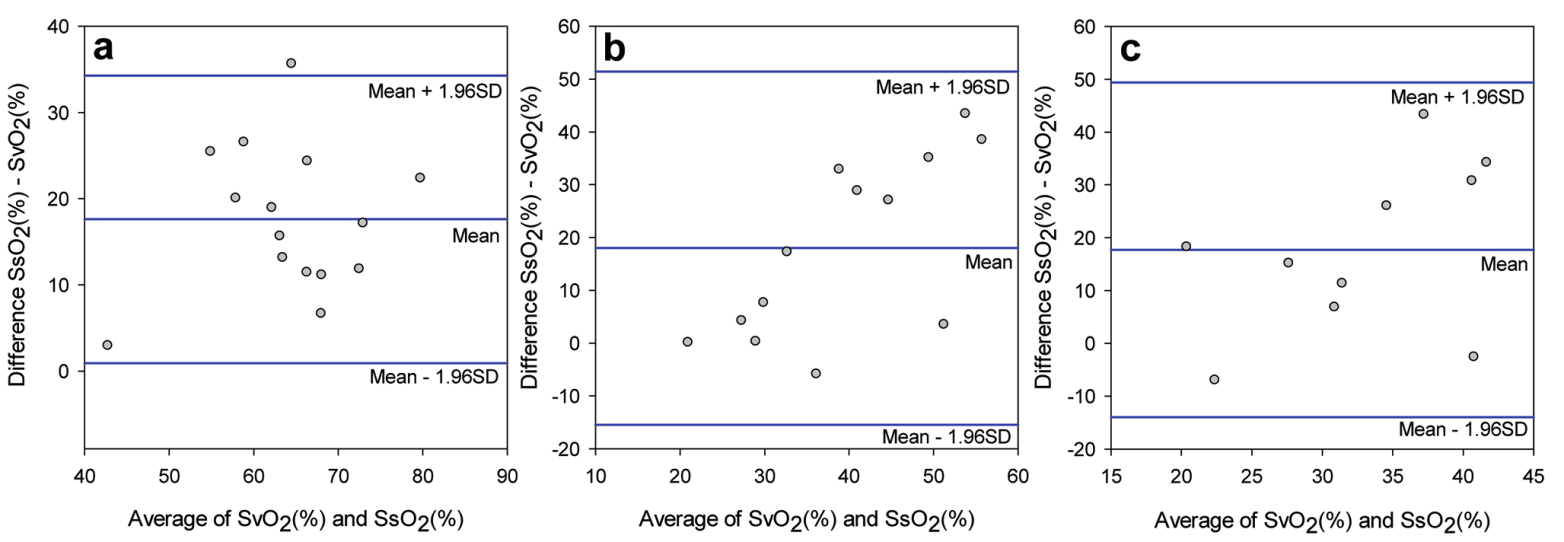
Bland-Altman plots illustrating the agreement between medullary sternal oxygen saturation (SsO_2_) and mixed venous oxygen saturation (SvO_2_) for (a) baseline, (b) hypovolaemia, and (c) hypoxia. Because of technical difficulties, data for SvO_2_ is missing for six animals during the hypoxic provocation

Agreement between SsO_2_ and SvO_2_ were analysed with Bland-Altman plots. The bias for baseline was 17.6 (SD 8.5), end of hypovolaemia 18.0 (SD 17.1) and at hypoxia 17.7 (SD 16.2), respectively. There was a consistent bias for the three situations but with a low precision according to large confidence intervals (Fig. 5).

### Haemorrhage

**Fig. 6:**
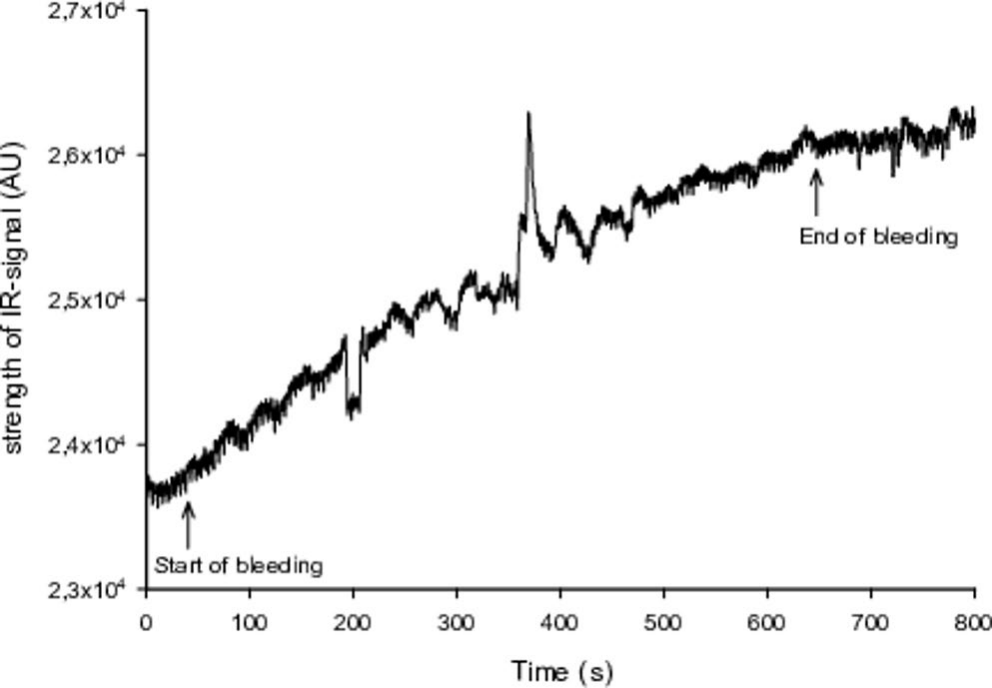
Illustration of the time course during the hypovolaemic provocation for one selected individual. The intensity of the infrared (IR)-wavelength, from the non-invasive sternal probe, deviates from baseline at the onset of bleeding (40 s) and plateaus when the target MAP has been reached, and the exsanguination ceases (640 s)

**Fig. 7:**
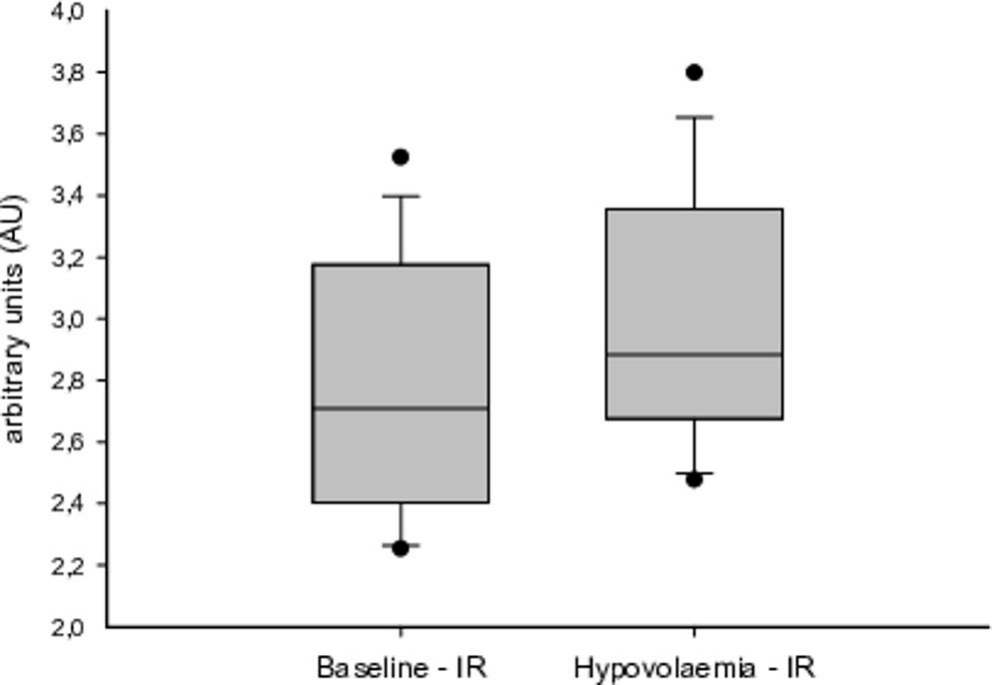
A box plot illustrating the increase in signal strength of the infrared (IR)-wavelength, from the sternal probe, during concomitant exsanguination

Signal artefacts in the sternal probe reading during hypovolaemia prevented further analysis in one animal. Hence, individual analyses were performed using 15 animals. Figure 6 is a typical registration of the IR-wavelength, from the sternal probe, for one selected individual. The start of positive inclination marks the onset of bleeding, which subsequently ends on a plateau where the target MAP is reached, and the rate of bleeding is drastically reduced. In Fig. 7, a boxplot shows the dynamic distribution of how the signal strength from the IR-wavelength is altered during bleeding. The registered intensity of the IR-wavelength had a significant increase from baseline with a mean difference of 0.23 Arbitrary units (AU) (SD 0.024), p < 0.001.

## Discussion

The main finding of the current study is that sternal medullary oxygen saturation has a value that is lower than arterial but higher than mixed venous oxygen saturation, indicating an arteriovenous mixture of blood. When capillary extraction of oxygen was increased as the effect of hypovolaemia, SaO_2_ was, as expected, unaltered. However, SsO_2_ changed with SvO_2_ found in the pulmonary artery. Changes in the sternal probe readings relate to changes in SsO_2_, and thus SvO_2_, during hypovolaemia or hypoxia.

The anatomical structure of bone tissue results in mixed arteriovenous blood flow [20]. Even though the sternum has a sustained haematopoietic activity and is highly vascularised [21], the SsO_2_ has been estimated to be roughly between 70 and 80% [13, 15]. With the assumption that the sternum has abundant blood perfusion, we have earlier shown the feasibility of monitoring arterial oxygen changes induced by hypoxia with a non-invasive probe targeting the sternal intraosseous portion of blood [11]. A linear association between SQV and SaO_2_, in that study, made it possible to calibrate SQV against SaO_2_ and hence estimate arterial oxygen saturation, despite a lower oxygen saturation in the marrow. However, these saturation changes were performed at rest and during normovolaemia. Little is known to which extent sternal perfusion is altered when the total circulating blood volume is reduced and the impact of intramedullary oxygen extraction on SsO_2_.

During exsanguination, SaO_2_ remained unchanged, while a decrease in both SvO_2_ and SsO_2_ was noted (Table 1; Fig. 2a). This was as expected since a reduction in tissue oxygen delivery leads to increased extraction of oxygen and hence a reduction of oxygen saturation in venous blood. Contrary to the hypoxic provocation, which demonstrated a significant decrease in SsO_2_, SaO_2_ and SvO_2_. Although not as profound as in the hypoxic experiments, the resulting decrease in SsO_2_ during hypovolaemia was detected by the sternal probe (Fig. 3). The resulting reduction of SsO_2_ after a decrease in FiO_2_ was accurately registered by the sternal probe (Figs. 2b, 3 and 4b), a result in line with previous findings. This implies that the blood oxygen saturation measured by non-invasive monitoring over the sternum is an arteriovenous mix. During circumstances with parallel changes in arterial and venous oxygen saturation, it appears possible to estimate arterial oxygen saturation from the sternal probe reading. However, this is probably not the case during situations with an increased sternal intramedullary oxygen extraction and maintained arterial oxygen saturation.

Oxygen deprived blood returning from the upper and lower part of the body mixes in the heart´s right ventricle before being re-saturated with oxygen in the lungs. This mixture of venous blood is referred to as mixed venous blood, and its saturation (SvO_2_) reflects the global oxygen consumption and delivery in the body. SvO_2_ is usually between 65 and 85% in both pigs and humans [22, 23]. A low SvO_2_ generally represents either a state of decreased oxygen delivery (e.g. haemorrhage, hypoxia or reduced perfusion) or a high oxygen demand (e.g. hyperthermia, pain or shivering). On the contrary, a high SvO_2_ indicates either an inability to use oxygen, e.g. in sepsis, high-flow states or a decreased oxygen demand (e.g. hypothermia or anaesthesia) [24–26]. SvO_2_ is clinically a very valuable indicator of adequate oxygen status [23, 25, 27, 28]. The downside is the need for central venous cannulation and insertion of a pulmonary artery catheter (PAC), a procedure not without risks [29]. A less invasive alternative to placing a PAC is to analyse central venous oxygen saturation (ScvO_2_) from the superior vena cava as a surrogate for SvO_2_, as the trend differences are the same for the two [30]. A possibility for non-invasive estimation of SvO_2_ would be of value for clinical decision making. Especially since deviations of normal SvO_2_-values has been linked as a marker for clinical outcome in a variety of situations, e.g. mortality in septic patients, postoperative complications or the success of weaning a patient from a ventilator [31–36]. Apart from PPG, Near infrared Spectroscopy (NIRS) is another optical method capable of measuring regional tissue oxygenation by determining the concentrations of oxygenated and deoxygenated haemoglobin [37–39]. The use of NIRS has been evaluated in several studies focusing on central or mixed venous oxygen saturation [30, 40–42]. In our study, SQV and SvO_2_ changed in parallel during blood loss, whereas SaO_2_ remained unaltered, indicating the potential for non-invasive measurement of SvO_2_ over the sternum. Although SvO_2_ generally was lower than the SsO_2_, the bias between SsO_2_ and SvO_2_ in Bland-Altman plots (Fig. 5) was consistent for baseline, hypoxia and hypovolaemia. One possible solution for this is using a conversion factor to the measured SQV to estimate the SvO_2_. However, like calibrating a device for measuring SaO_2_, this sternal probe will need to be calibrated against a spectrum of various mixed venous oxygen saturations to give a continuous accurate estimate of SvO_2_.

Using photoplethysmography (PPG) for assessing hypovolaemia has been a subject of extensive research, with varying results [43–47]. The DC-component of the PPG-signal has been assumed to represent total illuminated blood volume and thereby an indirect estimate of average circulating blood volume [10]. This study found that the signal strength of the IR-wavelength, from the sternal probe, increased almost momentarily as the exsanguination was started (Fig. 6). One explanation for this might be that a reduced blood volume will absorb less light and then more light will reach the photodetector, which is in line with previously reported observations [48].

### Limitations

The current results derive from animal experiments and cannot be directly applied to humans. However, inducing profound circulatory and respiratory effects in human volunteers is unethical and reinforces the need for animal testing. We used a porcine experimental model, as we had vast previous experience in large animal experiments. The size of the animals chosen is convenient for easy handling and enables cannulation of all major vessels. In addition, the physiology is similar to humans and well described [49].

In our study, the values of the SQV varied between individuals. There are numerous possible causes for the wide inter-individual variation. The anatomy of the pig sternum is different from the human sternum, and the sternal probe is not explicitly designed for porcine use. Also, the pig’s subcutaneous tissue is loose, and there were some issues securing the probe from displacement during the experiment. Reviewing the recordings has shown a tendency for motion artefacts, especially for the red wavelength.

Other possible limitations in the study were that the animals were young (mean weight of 25.8 kg) with incomplete ossification of the sternum. Also, the distribution and volume of intramedullary blood for this animal population is unclear. Despite this, there were only on a few occasions were intraosseous samplings was unsuccessful. Aspiration of sternal blood introduced, on some occasions, artefacts in sternal probe recording. These artefacts imply a functional communication of blood aspirated and the blood volume measured by the probe. To note, however, is that the study does not aim to evaluate invasive measurements of SsO_2_ in the clinical setting. Another limitation was that the batteries in the probe had insufficient capacity for a continuous recording of the entire experiment, resulting in several consecutive recordings during the times of interest. The possibility for drifts in baseline between the separate recordings cannot be excluded.

### Conclusion

This study further indicates that medullary blood flow in the sternum is a mixture of arterial and venous blood, resulting in average medullary oxygen saturation slightly higher than SvO_2_. In addition, this investigation demonstrates that non-invasive estimation of reductions in SsO_2_ in the pig during hypovolaemia and hypoxia is feasible. During pure hypoxia, this measurement reflects changes in both SaO_2_ and SvO_2_. When peripheral oxygen delivery is decreased as the effect of hypovolaemia, alterations in SvO_2_ are reflected by this technique. However, the estimation of SaO_2_ is flawed. The significant signal strength elevation, of the IR-wavelength, during the exsanguination suggests a possibility for non-invasive monitoring of volaemic status.
